# A Method Used to Improve the Dynamic Range of Shack–Hartmann Wavefront Sensor in Presence of Large Aberration

**DOI:** 10.3390/s22197120

**Published:** 2022-09-20

**Authors:** Wen Yang, Jianli Wang, Bin Wang

**Affiliations:** 1 Changchun Institute of Optics, Fine Mechanics and Physics, Chinese Academy of Sciences, Changchun 130033, China; 2 University of Chinese Academy of Sciences, Beijing 100049, China

**Keywords:** shack–hartmann wavefront sensor, autocorrelation method, neural network, dynamic range

## Abstract

With the successful application of the Shack–Hartmann wavefront sensor in measuring aberrations of the human eye, researchers found that, when the aberration is large, the local wavefront distortion is large, and it causes the spot corresponding to the sub-aperture of the microlens to shift out of the corresponding range of the sub-aperture. However, the traditional wavefront reconstruction algorithm searches for the spot within the corresponding range of the sub-aperture of the microlens and reconstructs the wavefront according to the calculated centroid, which leads to wavefront reconstruction errors. To solve the problem of the small dynamic range of the Shack–Hartmann wavefront sensor, this paper proposes a wavefront reconstruction algorithm based on the autocorrelation method and a neural network. The autocorrelation centroid extraction method was used to calculate the centroid in the entire spot map in order to obtain a centroid map and to reconstruct the wavefront by matching the centroid with the microlens array through the neural network. This method breaks the limitation of the sub-aperture of the microlens. The experimental results show that the algorithm improves the dynamic range of the first 15 terms of the Zernike aberration reconstruction to varying degrees, ranging from 62.86% to 183.87%.

## 1. Introduction

The Shack–Hartmann wavefront sensor (SHWFS) has achieved a wide range of well-established applications in adaptive optics systems due to its compact structure, high utilization of luminous energy, and ability to operate continuous or pulsed target light [1,2,3,4]. An SHWFS consists of a microlens array and a photodetector (currently mostly CCDs) [5,6]. If there is a phase distortion in the incident wavefront, the spot formed by each lenslet deviates from its focal plane. The sub-aperture spot offset between the measured wavefront and the reference wavefront reflects the instantaneous average wavefront slope of the incident wavefront within the sub-aperture, and the average slope in the x and y quadrature directions within the sub-aperture can be obtained via computer processing. The incident wavefront phase can be reconstructed based on the average slope of each sub-aperture [7,8]. From the working principle of SHWFS, it can be seen that the wavefront detection accuracy mainly depends on the centroid detection accuracy of the spot corresponding to each sub-aperture.

To date, a large number of studies have focused on improving the center of gravity (CoG) method [9,10,11,12,13,14,15,16], but, if the aberration to be measured is too large and the fluctuation of the wavefront is too steep, it will cause some sub-aperture spots to deviate from the corresponding sub-aperture. This condition is commonly measured in patients with severe refractive errors and patients who have undergone corneal or lens surgery [17,18]. Carmen Canovas and Erez N. Ribak compared and analyzed SHWFS methods (convolution, interpolation, Fourier methods, and centroid methods) for ophthalmology, and found that the Fourier method has the best effect for pupils with a small slope of the boundary or with a large distance from the boundary [19]. In the field of optical detection, such as the surface shape detection of high-order parabolic mirrors and conventional optical elements, it is important to reduce the measurement error by overcoming the problem of steep or linear edges [20,21,22,23,24]. In addition, researchers are focused on trying to rectify the distortion caused by the atmosphere to the propagating laser beam, which may detect optical vortices [25,26,27,28].

On the one hand, the traditional wavefront reconstruction algorithm attempts to find the spot within the corresponding range of the microlens sub-aperture and reconstructs the wave surface according to the calculated centroid displacement. When the partial wavefront distortion is large, the spot corresponding to the microlens shifts out of the sub-aperture range, and the traditional algorithm cannot search for the spot. In this case, the focal coordinate of the microlens is usually taken as the calculated spot centroid, which leads to the local large distortion being miscalculated as a plane wave; thus, the wavefront reconstruction is incorrect. On the other hand, when the spot enters the other adjacent sub-aperture range, the traditional algorithm takes the common centroid of two spots as the centroid of the current sub-aperture spot, which also leads to wavefront reconstruction errors. Although the dynamic range of the wavefront sensor can be effectively increased by reducing the focal length of the microlens, the centroid detection accuracy of the wavefront sensor will be reduced, which is not worth the gain. Therefore, it is necessary to eliminate the limitation of the microlens sub-aperture on the spot in traditional wavefront reconstruction algorithms.

To expand the dynamic range of SHWFS, C. Leroux proposed an iterative extrapolation method based on measuring the centroid positions [29]. J. Pfund used the improved expansion algorithm to realize that, as long as the change in the spot dislocation between two adjacent sub-apertures is less than half of the distance between the two sub-apertures, it is possible to clearly allocate the sub-apertures of each spot [30]. Geun-Young Yoon used a translatable plate of sub-apertures placed conjugate to the lenslet arrays [31]. Norbert Lindlein solved this problem by using a spatial light modulator array in front of the microlens of the sensor to control the sub-aperture opening or closing [32]. Zeyu Gao proposed a centroid estimation algorithm based on image segmentation and related techniques to expand the dynamic range based on search strategies [33]. Meanwhile, artificial intelligence methods have been widely used in Shack–Hartmann wavefront sensing technology. For example, the strong fitting ability of neural networks is used for wavefront restoration [34,35], and, even when the sub-aperture is sparse [36,37] or in an extremely low signal–noise ratio (SNR) situation [38], it can still work well. Additionally, in the multi-sensor data fusion field [39,40], deep learning methods have also shown their effectiveness.

This paper presents a new wavefront reconstruction algorithm used to expand the dynamic range. Based on the improved autocorrelation centroid detection method, the centroid of the spot is calculated from the entire spot image, and the correlation between the centroid of the spot and the microlens is transformed into a classification problem using a neural network to reconstruct the wavefront. Not only does our method eliminate the limitation of the microlens sub-aperture on the spot, but it also does not need to add other auxiliary conditions, such as hardware; even if the local wavefront aberration is large, the spot is offset out of the corresponding microlens aperture or even into the adjacent sub-aperture, and the algorithm can still accurately reconstruct the wavefront. We verified the accuracy of the method using a numerical simulation. Compared with the traditional method, the dynamic range of the different terms of the Zernike polynomials is improved by 62.86~183.87%.

## 2. Methods

### 2.1. Shack–Hartmann Wavefront Sensing Technology

**Figure 1:**
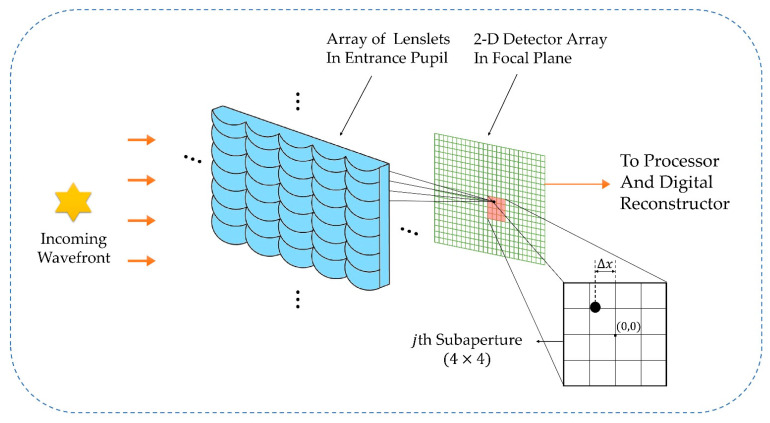
Schematic diagram of the SHWFS principle.

As the core device of Shack–Hartmann wavefront sensing technology, an SHWFS is composed of a microlens array and a CCD camera [41,42], as shown in Figure 1. The microlens array converts the wavefront information into tilt information, which is collected by CCD, and then the wavefront is restored via computer operation. The entire process of this technology includes three steps: centroid calculation, slope calculation, and wavefront reconstruction. In the wavefront reconstruction of an SHWFS, the wavefront error information of the full aperture is mainly detected according to the calculated position of the spot $\left(x_{i},y_{i}\right)$:

(1) $$\left\{\begin{array}{l} x_{i}=\frac{\sum_{m=1}^{M}\sum_{n=1}^{N}x_{nm}I_{nm}}{\sum_{m=1}^{M}\sum_{n=1}^{N}I_{nm}}\\ y_{i}=\frac{\sum_{m=1}^{M}\sum_{n=1}^{N}y_{nm}I_{nm}}{\sum_{m=1}^{M}\sum_{n=1}^{N}I_{nm}} \end{array}\right.$$

where the detection region of each sub-aperture focal plane is M×N, m=1∼M and n=1∼N are the sub-apertures mapped to the corresponding pixel region on the CCD photosensitive target surface, and $I_{nm}$ is the signal received by the $\left(n,m\right)$ pixel on the CCD photosensitive target surface. $x_{nm}$ and $y_{nm}$ are the X and Y coordinates of the $\left(n,m\right)$ pixel, respectively.

Then, the wavefront slope of the incident wavefront is calculated according to the following formula:

(2) $$\left\{\begin{array}{l} g_{xi}=\frac{\Delta x}{f}=\frac{x_{i}-x_{0}}{f}\\ g_{yi}=\frac{\Delta y}{f}=\frac{y_{i}-y_{0}}{f} \end{array}\right.$$

where $\left(x_{0},y_{0}\right)$ is the centroid coordinate of the spot array image formed by the calibrated wavefront, $\left(x_{i},y_{i}\right)$ is the centroid coordinate of the spot array image formed by the measured wavefront, i is the corresponding sub-aperture serial number, and f is the focal length of the microlens array.

To convert the slope information measured by an SHWFS into the phase of the wavefront or the voltage value of the deformable mirror driver, an algorithm, which is called the wavefront reconstruction algorithm, is required to establish the relationship between the slope and the phase/driving voltage. The most widely used methods are the region method [43,44,45], the model method [46], and the direct slope method [47]. Among them, the mode reconstruction method based on the Zernike polynomial is widely used in wavefront detection because it can restore the continuous wavefront phase. This method is also used in this paper for wavefront reconstruction. The basic principle is to describe the phase to be measured in the circular domain with a set of Zernike polynomials [48]:

(3) $$\phi \left(x,y\right)=a_{0}+\sum_{k=1}^{n}a_{k}Z_{k}\left(x,y\right)+\varepsilon$$

where $a_{0}$ is the average wavefront phase, $a_{k}$ is the coefficient of the k-term Zernike polynomial $Z_{k}\left(x,y\right)$, n is the mode order, and ε is the residual phase measurement error.

For the i-th sub-aperture, the average slopes $G_{x}\left(i\right)$ and $G_{y}\left(i\right)$ within the sub-aperture are the average of the gradients of the wavefront phase in the x and y directions, respectively:

(4) $$\left\{\begin{array}{l} G_{x}\left(i\right)=\sum_{k=1}^{n}a_{k}\frac{\iint_{S_{i}}\frac{\partial Z_{k}\left(x,y\right)}{\partial x}dxdy}{S_{i}}+\varepsilon_{x}\\ G_{y}\left(i\right)=\sum_{k=1}^{n}a_{k}\frac{\iint_{S_{i}}\frac{\partial Z_{k}\left(x,y\right)}{\partial y}dxdy}{S_{i}}+\varepsilon_{y} \end{array}\right.$$

where $\varepsilon_{x}$ and $\varepsilon_{y}$ are the residual phase measurement errors, and $S_{i}$ is the normalized area of the sub-aperture.

Order $Z_{xk}\left(i\right)=\frac{\iint_{S_{i}}\frac{\partial Z_{k}\left(x,y\right)}{\partial x}dxdy}{S_{i}}$, $Z_{yk}\left(i\right)=\frac{\iint_{S_{i}}\frac{\partial Z_{k}\left(x,y\right)}{\partial y}dxdy}{S_{i}}$; then, there are

(5) $$\left\{\begin{array}{l} G_{x}\left(i\right)=\sum_{k=1}^{n}a_{k}Z_{xk}\left(i\right)+\varepsilon_{x}\\ G_{y}\left(i\right)=\sum_{k=1}^{n}a_{k}Z_{yk}\left(i\right)+\varepsilon_{y} \end{array}\right.$$

For an SHWFS with m effective sub-apertures, when restoring the phase represented by n-term Zernike polynomials, the relationship between the slope of the sub-aperture and the Zernike coefficient is expressed as a matrix:


(6) $$\left[\begin{array}{c} G_{x}\left(1\right)\\ G_{y}\left(1\right)\\ G_{x}\left(2\right)\\ G_{y}\left(2\right)\\ \cdots \\ G_{x}\left(m\right)\\ G_{y}\left(m\right) \end{array}\right]=\left[\begin{array}{cccc} Z_{x1}\left(1\right) & Z_{x2}\left(1\right) & \cdots & Z_{xn}\left(1\right)\\ Z_{y1}\left(1\right) & Z_{y2}\left(1\right) & \cdots & Z_{yn}\left(1\right)\\ Z_{x1}\left(2\right) & Z_{x2}\left(2\right) & \cdots & Z_{xn}\left(2\right)\\ Z_{y1}\left(2\right) & Z_{y2}\left(2\right) & \cdots & Z_{yn}\left(2\right)\\ \cdots & \cdots & & \cdots \\ Z_{x1}\left(m\right) & Z_{x2}\left(m\right) & \cdots & Z_{xn}\left(m\right)\\ Z_{y1}\left(m\right) & Z_{y2}\left(m\right) & \cdots & Z_{yn}\left(m\right) \end{array}\right]\left[\begin{array}{l} a_{1}\\ a_{2}\\ \cdots \\ a_{n} \end{array}\right]+\left[\begin{array}{c} \varepsilon_{1}\\ \varepsilon_{2}\\ \varepsilon_{3}\\ \varepsilon_{4}\\ \cdots \\ \varepsilon_{2m-1}\\ \varepsilon_{2m} \end{array}\right]$$

This matrix can be written as

(7) G=DA+ε

where G is the slope vector, D is the mode coefficient reconstruction matrix, and A is the corresponding Zemike coefficient.

At this time, the wavefront reconstruction process can be regarded as a solution process for the above-mentioned linear equations. The least-squares solution of Equation (7) can be obtained by measuring the slope vector G:

(8) $$A=D^{+}G$$

where $D^{+}$ is the pseudo-inverse of D, which can be solved by a singular value decomposition (SVD).

Therefore, when the phase is measured after the calibration is completed, the Zernike polynomial coefficient A can be obtained as long as the slope G of each sub-aperture is calculated, and the phase to be measured can be reconstructed by Equation (3).

### 2.2. Centroid Detection Based on Autocorrelation Method

Sub-spot localization is the first step for an SHWFS to reconstruct the wavefront. By analyzing the shape of the spot distribution detected by an SHWFS, it is found that the actual spot distribution is approximately normal [49]. This is because the illumination area of the detection light on the retina is approximately circular, and when it reaches the CCD array of the wavefront sensor through the retinal imaging system, it is still approximately circular. Although the light spot detected by the CCD is affected by the aberrations of the human eye, the aberrations of the human eye mainly show defocus and astigmatism; the higher order aberration component is relatively small, and the defocus aberration and astigmatism are both central symmetric. Therefore, it is reasonable to approximate the actual spot distribution to the normal distribution. Based on this premise, we introduced the autocorrelation method into the centroid detection algorithm. Through the convolution operation of the signal and the autocorrelator template, the response value was obtained. When the amplitude spectrum of the signal is consistent with the amplitude characteristics of the autocorrelator template, the response is the largest. According to the convolution characteristics of Fourier transform, the convolution in the time domain can be expressed as the product in the frequency domain, and the autocorrelator response can be expressed as

(9) $$CR\left(x,y\right)=FT^{-1}\left\{FT\left\{\left.I\left(X,Y\right)\right\}\right.\right.\times FT\left\{\left.H\left(X,Y\right)\right\}\right.$$

where $CR\left(x,y\right)$ represents the matched filter response, FT represents the Fourier transform, $I\left(X,Y\right)$ represents the spot pattern, and $H\left(X,Y\right)$ represents the autocorrelator template.

The key to the autocorrelation method is determining how to select an autocorrelator template that is consistent with the amplitude characteristics of the target signal. For a retinal adaptive imaging system, according to the parameters of the optical system and the distribution pattern of the illumination light during the aberration detection of the human eye, the distribution pattern of the spot in the wavefront sensor can be calculated as the autocorrelator template. Due to the dynamic characteristics of the human eye aberration analyzed above, the influence of the human eye aberration can be ignored when selecting the autocorrelator template, and the amplitude spectrum of the actual spot can also be well estimated. 

In practical application, we calculated the convolution response of the actual spot to the autocorrelator template with different center coordinates, and we took the center coordinate of the autocorrelator template at the maximum value of the response as the calculated spot centroid:

(10) $$\left(x_{i},y_{i}\right)=\arg\max CR\left(x,y\right)$$

When the centroid position of the spot is found, the local area of the spot in the correlation matrix resets to zero. Then, the next spot centroid position was calculated until all k centroids in the spot array were traversed (*k* is the number of effective microlens sub-apertures).

**Figure 2:**
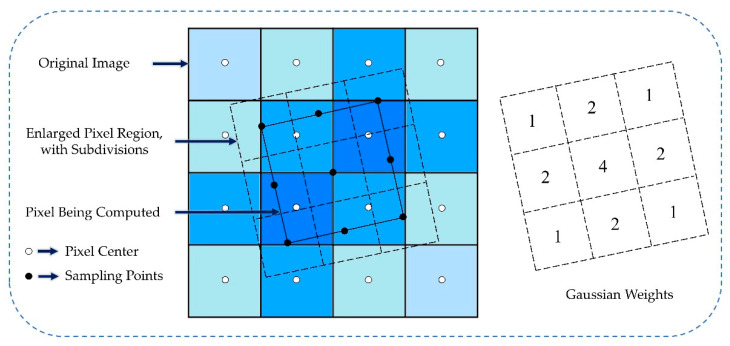
Schematic diagram of Gaussian-weighted bilinear interpolation method.

The autocorrelation algorithm has the disadvantage of the error being greatly affected by the sampling frequency. In order to maintain the accuracy of centroid detection, it is necessary to ensure that the processed pixels are small enough. In this case, the Gaussian-weighted bilinear interpolation method was used to determine the centroid position of the spot by using a small window around the maximum value of the correlation response. As shown in Figure 2, the interpolation algorithm works by subsampling the pixels to obtain the sub-pixels and then by performing bilinear interpolation to obtain their respective gray values; each sub-pixel has different weights when contributing its gray values. For a Gaussian distribution spot, the corresponding Gaussian distribution template is adopted.

**Figure 3:**
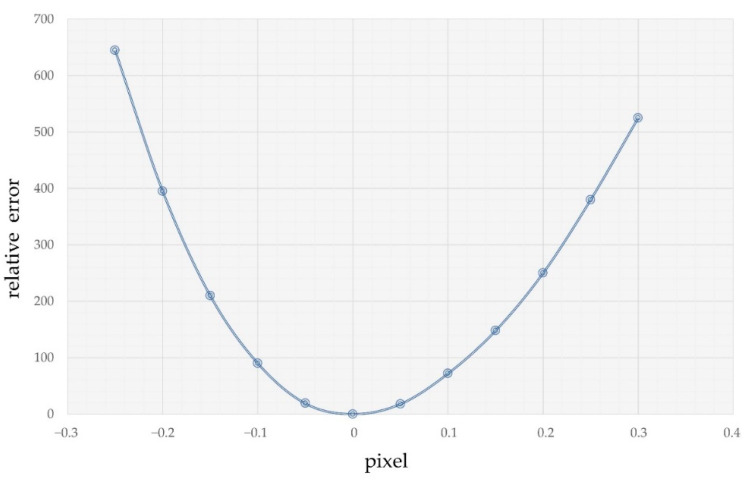
Relative error corresponding to template movement near the centroid of the spot.

When the template moves near the actual centroid of the spot without the influence of noise or multi-layer reflected stray light, the relative error between the template and the spot distribution is shown in Figure 3; here, only the abscissa direction is considered. From the error curve in Figure 3, it is found that the relative error is minimum when the center of the autocorrelation template coincides with the center of the spot, and the relative error increases with the distance of the autocorrelation template from the center of the spot. Here, it is assumed that the autocorrelation template parameters calculated according to the optical system parameters coincide with the Gaussian distribution spot parameters. Therefore, when the autocorrelation template center coincides with the spot centroid, the relative error is 0.

Because the centroid detection algorithm based on the autocorrelation method has the advantage of calculating the centroid position in the entire spot array, rather than in the corresponding region of a certain sub-aperture, the wavefront can still be reconstructed effectively by matching the calculated spot centroid with the microlens one by one with the corresponding algorithm. Therefore, the algorithm can also be used to expand the dynamic range of an SHWFS. The processing speed of the autocorrelation method is 33 fps, which basically meets the real-time requirements. In the next section, we discuss in detail a new spot-matching algorithm based on the above detection algorithm to expand the dynamic range of SHWFS.

### 2.3. Spot-Matching Network

**Figure 4:**
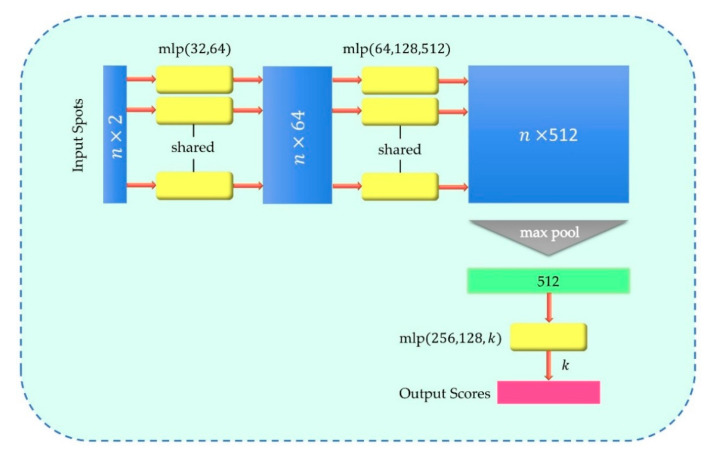
Spot-matching neural network structure.

We consider the problem of matching the spot with the sub-aperture as a classification problem. Firstly, the microlens array is numbered in the form of one-hot to facilitate neural network training. Then, the preprocessed centroid coordinates and labels of each sub-aperture spot are used as the input layer, which is propagated forward through the hidden layer. Finally, the predicted probability of each sub-aperture of the spot is generated by the output layer. The network structure is shown in Figure 4.

**Figure 5:**
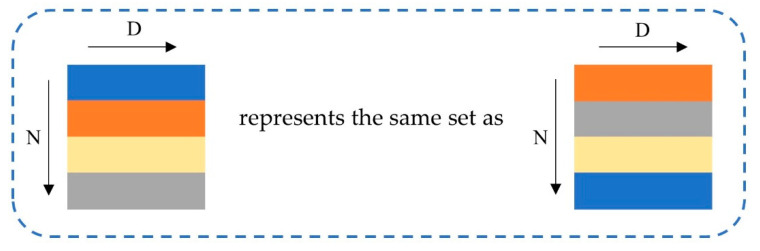
Disorder characteristics of data.

We observed that the input of the neural network is the centroid coordinate point, which is a two-dimensional coordinate set and has disordered characteristics. Specifically, as Figure 5 shows, the order of the centroid coordinate data does not affect the position or the property of the point on the spot map; that is, it is not sensitive to the order of the data. This means that the model processing the centroid coordinate data needs to maintain invariance to the different arrangements of the data.

For the disorder problem, we adopted the design of symmetry functions,

(11) $$f\left(x_{1},x_{2},\ldots ,x_{n}\right)\equiv f\left(x_{\pi_{1}},x_{\pi_{2}},\ldots ,x_{\pi_{n}}\right),x_{i}\in {\mathbb{R}}^{D}$$

For example, Sum and Max are common symmetric functions.

(12) $$f\left(x_{1},x_{2},\ldots ,x_{n}\right)\equiv \max\left\{x_{1},x_{2},\ldots ,x_{n}\right\}$$

(13) $$f\left(x_{1},x_{2},\ldots ,x_{n}\right)\equiv x_{1}+x_{2}+\ldots +x_{n}$$

Although the direct symmetry operation on the data satisfies the permutation invariance, it is easy to lose a large amount of geometric and meaningful information. For example, when taking the maximum value, only the farthest point is obtained; when taking the average value, only the center of gravity is obtained. For the expression of two-dimensional points in a high-dimensional space, information must be redundant. To reduce the loss of information, we mapped each point to a high-dimensional space and performed symmetry operations on the data in the high-dimensional space so that we could retain enough point information.

Here, a symmetric function takes n vectors as the input and outputs a new vector independent of the input order to approximate a general function defined on the point set:

(14) $$f\left(\left\{\left.x_{1},\ldots ,x_{n}\right\}\right.\right)\approx g\left(h\left(x_{1}\right),\ldots ,h\left(x_{n}\right)\right)$$

where $f:2^{{\mathbb{R}}^{N}}\to \mathbb{R}$, $h:{\mathbb{R}}^{N}\to {\mathbb{R}}^{K}$, $g:\underset{n}{\underbrace{{\mathbb{R}}^{K}\times \cdots \times {\mathbb{R}}^{K}}}\to \mathbb{R}$ is a symmetric function.

**Figure 6:**
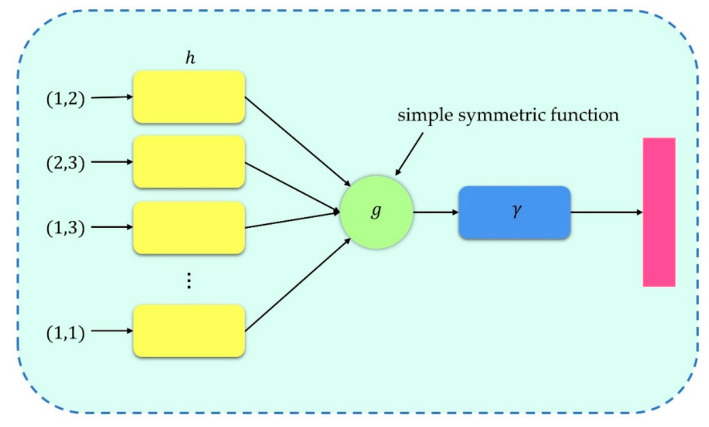
Basic modules of the neural network.

As shown in Figure 6, our basic module is simple: we approximate with a multi-layer perceptron (MLP) network and with a combination of a single-variable function and a max pooling function. In this way, after the network performs a certain degree of feature extraction on each point, the global feature can be extracted from the overall points through max pooling. In our network, MLP was realized by convolution with shared weights. The size of the first layer convolution kernel was 1×2 (because the dimensions of each point are X and Y), and each subsequent convolution kernel size was 1×1. After two MLPs, 512-dimensional features were extracted for each point, and then they were transformed into 1×512 global features through max pooling. After the last MLP, the output was classification scores for k classes. All layers, except for the last one, included ReLU and batch normalization.

The loss function used in the network was the softmax loss. The formula is as follows:

(15) $$Loss=-\sum_{i=1}^{N}y_{i}\log{\hat{y}}_{i}$$

where N is the number of the samples, $y_{i}$ is the real sample label, and ${\hat{y}}_{i}$ is the prediction label. The loss function represents the difference between the forward calculation result of each iteration of the neural network and the real value. The smaller the loss function, the more accurate the network classification.

## 3. Results

### 3.1. Data Preparation and Implementation Details

To verify the effectiveness of the algorithm, we carried out a series of numerical simulation experiments. Table 1 shows the key parameters of the simulation. The incident wavefront was derived from the first 15 Zernike polynomials (excluding piston and tilt), because the first 15 Zernike polynomials are sufficient for general wavefront sensing problems [50]. In addition, the Zernike coefficients were obtained from the randomly weighted Karhunen–Loève [51] atmospheric turbulence model. To enrich the dataset and test the performance of the new algorithm, we randomly generated 10,000 wavefronts. We randomly disrupted the order of the datasets, and we selected 9000 sets of centroid coordinates with their sub-aperture labels as the training set and the remaining 1000 sets as the test set. The noise of the sensor was not considered in the simulation.

The experimental environment was Intel(R)Core^TM^i7−9700 K CPU@3.60 GHz, DDR4 RAM 16 G, Windows 10, NVIDIA GEFORCE RTX2070S GPU. We performed network training in Pytorch 1.3 (created by FAIR, Menlo Park, CA, USA). We used the Adam optimizer as our optimization algorithm. Other experimental details about the neural network training are shown in Table 2.

### 3.2. Evaluation Indicators

The dynamic range is one of the most important metrics for wavefront sensors, and we chose the maximum root mean square (*RMS*) as the dynamic range indicator. Zernike polynomials are orthogonal and linearly independent of each other, and the coefficients of Zernike polynomials are positively correlated with *RMS*. In view of these properties, the dynamic range can be measured by increasing the coefficients of the Zernike polynomials when the other terms of coefficients remain zero. The maximum *RMS* is regarded as the dynamic range when the error between the reconstructed wavefront and the actual wavefront is less than 1% of the threshold value. The improvement in the dynamic range is calculated as

(16) $$\delta_{RMS}=\frac{RMS_{\mathrm{ours}}-RMS_{\mathrm{classical}}}{RMS_{\mathrm{classical}}}\times 100\%$$

where $RMS_{\mathrm{ours}}$ is the largest *RMS* of the wavefront measured by the proposed algorithm, and $RMS_{\mathrm{classical}}$ is the largest *RMS* of the wavefront measured by the classical algorithm.

### 3.3. Qualitative Results

**Figure 7:**
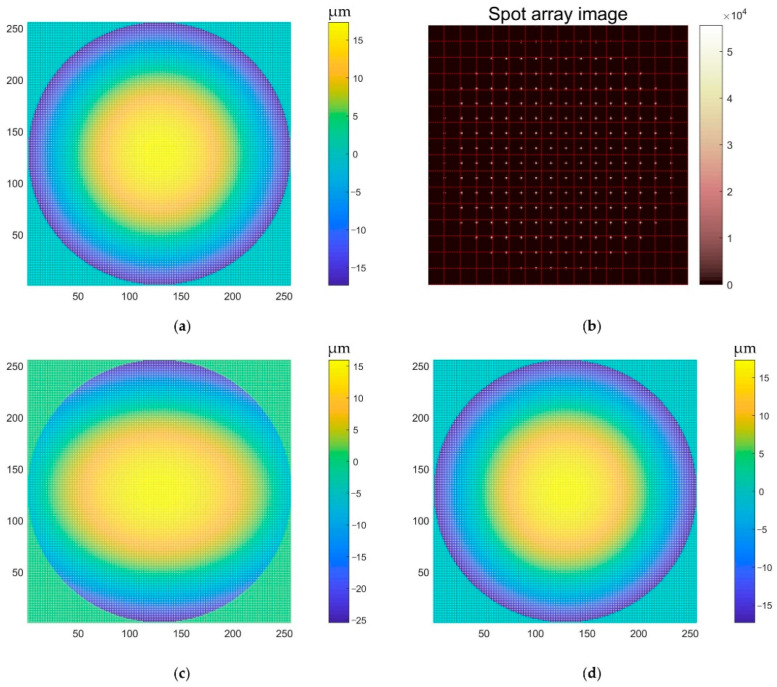
$Z_{2}^{0}$ (**a**) Reference wavefront; (**b**) spot array; (**c**) wavefront reconstructed with the classical algorithm; (**d**) wavefront reconstructed with our algorithm.

**Figure 8:**
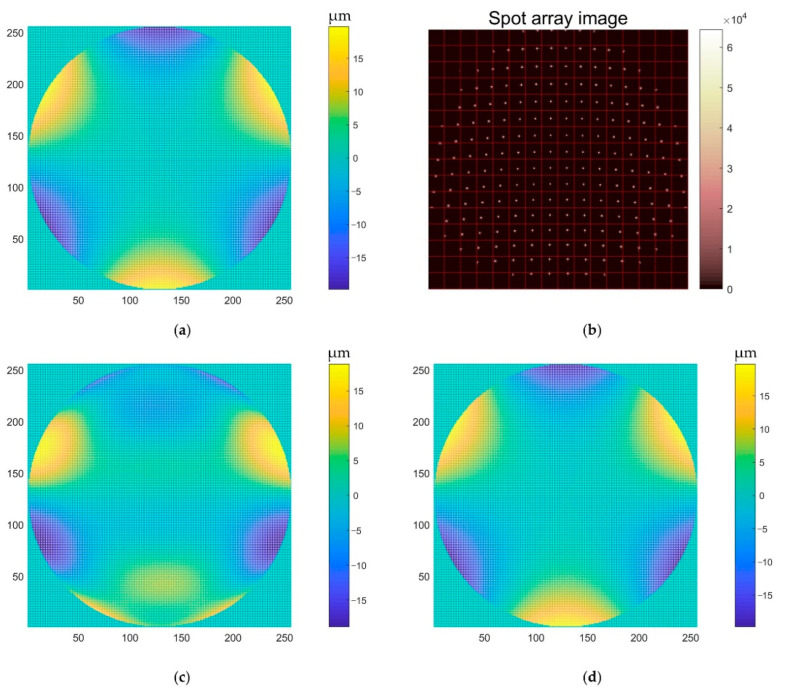
$Z_{3}^{-3}$ (**a**) Reference wavefront; (**b**) spot array; (**c**) wavefront reconstructed with the classical algorithm; (**d**) wavefront reconstructed with our algorithm.

To verify the effectiveness of the proposed algorithm, a comparative experiment with the traditional wavefront reconstruction algorithm was carried out. Since the local slope of the wavefront to be measured is related to the coordinates of the centroid of the spot, and, at the same time, the wavefront to be measured can be expressed in the form of Zernike polynomials, the corresponding spot centroid distribution can be calculated according to the wavefront corresponding to the Zernike coefficient. We used the traditional algorithm and our algorithm to reconstruct the wavefront, and we calculated the relative error of the reference wavefront corresponding to the Zernike coefficient. We gradually increased the Zernike coefficient and repeated the above process until the wavefront reconstructed by the two algorithms produced a large error. The results of $Z_{2}^{0}$ and $Z_{3}^{-3}$ are shown in Figure 7 and Figure 8, respectively. There are some spots in the spot array, part or all of which exceed the corresponding sub-aperture pixel region. At this moment, the classical algorithm cannot correctly reconstruct such a wavefront. However, our algorithm can reconstruct the correct wavefront information well.

### 3.4. Quantitative Results

**Figure 9:**
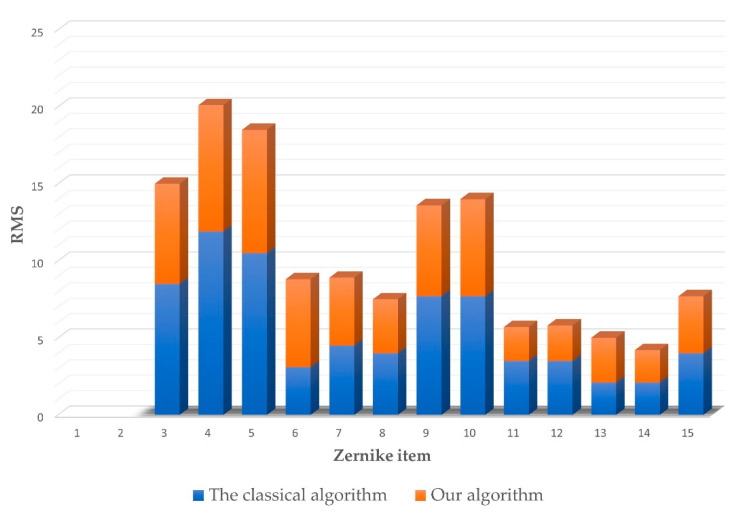
The dynamic range of the algorithms.

Through the numerical simulation, the dynamic range of the method was quantitatively evaluated. We calculated the dynamic range of our algorithm and the classical algorithm in the first 15 terms of Zernike polynomials without the piston and tilt. Compared with the classical algorithm, our algorithm improved the dynamic range of the Zernike polynomials by 62.86% to 183.87%. The result is shown in Figure 9. From the results, we can see that our method improves the dynamic range of low-order aberrations more obviously, which conforms to the basic characteristics of human eye aberration. For the higher-order aberrations, there is also a certain degree of improvement.

### 3.5. Limitation

**Figure 10:**
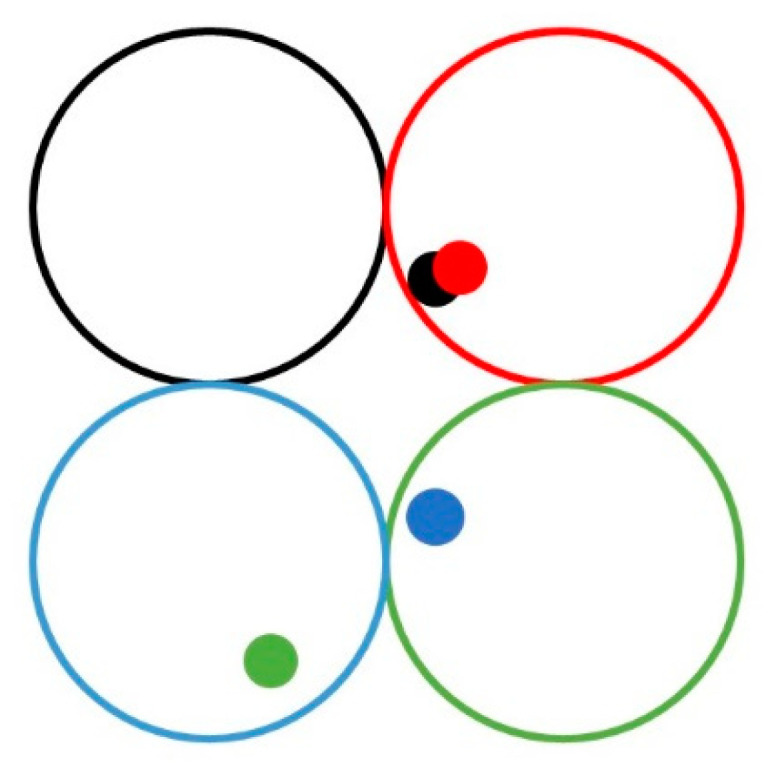
Schematic diagram of overlap and intersection of wavefront detection spots.

It should be noted that this method cannot handle two extreme cases. As shown in Figure 10, the wavefront detection spot is represented by the same color as the corresponding microlens, in which the black microlens and the spot of the red microlens are imaged in the adjacent pixel region of the CCD, and parts of the spot overlap with each other. At this time, it is impossible to distinguish whether the extended spot is caused by the overlap of two adjacent spots or by the aberration modulation of a single spot. Therefore, the local aberrations in this region cannot be calculated accurately. However, when the corresponding spot of the blue microlens enters the aperture range of the green microlens, and the corresponding spot of the green microlens enters the aperture range of the blue microlens, due to the limitations of the SHWFS’s principle, it is impossible to distinguish whether the microlens corresponding to the blue spot is the blue microlens or the green microlens; similarly, the green spot cannot be determined. The above two extreme cases inevitably lead to the wavefront reconstruction error, so the analysis of this paper is based on the spot distribution, which does not appear in the above extreme cases.

## 4. Conclusions

Due to the lack of a dynamic range, SHWFS has limited application in wavefront detection with large aberrations, such as human eye aberration detection. The traditional centroid algorithm and a large number of improved weighted centroid algorithms calculate the centroid of the light spot within the sub-aperture of the microlens, which cannot meet the requirements. The autocorrelation-based centroid algorithm used in this paper eliminates the limitation of the microlens sub-aperture in the traditional wavefront reconstruction algorithm without sacrificing the centroid detection accuracy, and it associates the spot centroid with the corresponding microlens through a neural network to reconstruct the wavefront. The comparative experimental results show that our algorithm can effectively improve the dynamic range of the wavefront sensor without adding any hardware facilities, and the dynamic range of Zernike’s first 15 aberrations can be improved from 62.86% to 183.87%.

## Figures and Tables

**Table 1:** The key parameters of the simulation.

Parameter	Value
Focal length of the lenslets	6.5 mm
Wavelength	500 nm
Lenslet numbers	16 × 16
Lenslet size	500 µm
Number of pixels in each sub-aperture	20 × 20 pixels
Pixel size	10 µm

**Table 2:** Training parameters.

Parameter	Value
Learning rate	0.001
Epoch	50
Batch size	8
Momentum	0.9
Decay rate	0.5~0.99

## Data Availability

Not applicable.

## References

[1] Ares J., Mancebo T., Bara S. (2000). Position and Displacement Sensing with Shack-Hartmann Wave-Front Sensors. Appl. Opt..

[2] Hartmann J. (1900). Bermerkungen über Den Bau Und Die Justierung Von Spektrographen. Z. Instrum..

[3] Shack R.V. (1971). Production and Use of a Lecticular Hartmann Screen. J. Opt. Soc. Am..

[4] Vargas J., González-Fernandez L., Quiroga J.A., Belenguer T. (2010). Shack–Hartmann Centroid Detection Method Based on High Dynamic Range Imaging and Normalization Techniques. Appl. Opt..

[5] Neal D.R., Copland J., Neal D.A. Shack-Hartmann Wavefront Sensor Precision and Accuracy. Proceedings of the Advanced Characterization Techniques for Optical, Semiconductor, and Data Storage Components.

[6] Primot J. (2003). Theoretical Description of Shack–Hartmann Wave-Front Sensor. Opt. Commun..

[7] Wang J.Y., Silva D.E. (1980). Wave-Front Interpretation with Zernike Polynomials. Appl. Opt..

[8] Soloviev O., Vdovin G. (2005). Hartmann-Shack Test with Random Masks for Modal Wavefront Reconstruction. Opt. Express.

[9] Thomas S., Fusco T., Tokovinin A., Nicolle M., Rousset G. (2006). Comparison of Centroid Computation Algorithms in a Shack–Hartmann Sensor. Mon. Not. R. Astron. Soc..

[10] Li X., Li X., Wang C. Optimum Threshold Selection Method of Centroid Computation for Gaussian Spot. Proceedings of the Aopc: Image Processing & Analysis.

[11] Lardière O., Conan R., Clare R., Bradley C., Hubin N. (2010). Compared Performance of Different Centroiding Algorithms for High-Pass Filtered Laser Guide Star Shack-Hartmann Wavefront Sensors. Proc. SPIE Int. Soc. Opt. Eng..

[12] Ma X., Rao C., Zheng H. (2009). Error Analysis of Ccd-Based Point Source Centroid Computation under the Background Light. Opt. Express.

[13] Leroux C., Dainty C. (2010). Estimation of Centroid Positions with a Matched-Filter Algorithm: Relevance for Aberrometry of the Eye. Opt. Express.

[14] Kong F., Polo M.C., Lambert A. (2017). Centroid Estimation for a Shack–Hartmann Wavefront Sensor Based on Stream Processing. Appl. Opt..

[15] Vargas J., Restrepo R., Estrada J.C., Sorzano C.O., Du Y.Z., Carazo J.M. (2012). Shack-Hartmann Centroid Detection Using the Spiral Phase Transform. Appl. Opt..

[16] Vargas J., Restrepo R., Belenguer T. (2014). Shack-Hartmann Spot Dislocation Map Determination Using an Optical Flow Method. Opt. Express.

[17] Schwiegerling J. (2014). History of the Shack Hartmann Wavefront Sensor and Its Impact in Ophthalmic Optics.

[18] van Ginkel R., Mechó M., Cardona G., González-Méijome J.M. (2022). The Effect of Accommodation on Peripheral Refraction under Two Illumination Conditions. Photonics.

[19] Canovas C., Ribak E.N. (2007). Comparison of Hartmann Analysis Methods. Appl. Opt..

[20] Zhao S., Cheng X. (2017). Application and Development of Wavefront Sensor Technology. Int. J. Mater. Sci. Appl..

[21] Sakharov A.M., Baryshnikov N.V., Karasik V.E., Sheldakova J.V., Kudryashov A., Nikitin A. A Method for Reconstructing the Equation of the Aspherical Surface of Mirrors in an Explicit Form Using a Device with a Wavefront Sensor. Proceedings of the Optical Manufacturing and Testing XIII.

[22] Rocktäschel M., Tiziani H.J. (2002). Limitations of the Shack–Hartmann Sensor for Testing Optical Aspherics. Opt. Laser Technol..

[23] Neal D.R., Pulaski P., Raymond T.D., Neal D.A., Wang Q., Griesmann U. Testing Highly Aberrated Large Optics with a Shack-Hartmann Wavefront Sensor. Proceedings of the Advanced Wavefront Control: Methods, Devices, and Applications.

[24] Li C. (2012). Three-Dimensional Surface Profile Measurement of Microlenses Using the Shack–Hartmann Wavefront Sensor. J. Micro Electromech. Syst..

[25] Sheldakova J., Kudryashov A., Zavalova V., Romanov P. Shack-Hartmann Wavefront Sensor Versus Fizeau Interferometer for Laser Beam Measurements. Proceedings of the Laser Resonators and Beam Control XI.

[26] Murphy K., Burke D., Devaney N., Dainty C. (2010). Experimental Detection of Optical Vortices with a Shack-Hartmann Wavefront Sensor. Opt. Express.

[27] Li T., Huang L., Gong M. (2014). Wavefront Sensing for a Nonuniform Intensity Laser Beam by Shack–Hartmann Sensor with Modified Fourier Domain Centroiding. Opt. Eng..

[28] Alexandrov A., Rukosuev A.L., Zavalova V.Y., Romanov P., Samarkin V.V., Kudryashov A.V. Adaptive System for Laser Beam Formation. Proceedings of the Laser Beam Shaping III.

[29] Leroux C., Dainty C. (2009). A Simple and Robust Method to Extend the Dynamic Range of an Aberrometer. Opt. Express.

[30] Pfund J., Lindlein N., Schwider J. (1998). Dynamic Range Expansion of a Shack–Hartmann Sensor by Use of a Modified Unwrapping Algorithm. Opt. Lett..

[31] Yoon G.-Y., Pantanelli S., Nagy L.J. (2006). Large-Dynamic-Range Shack-Hartmann Wavefront Sensor for Highly Aberrated Eyes. J. Biomed. Opt..

[32] Lindlein N., Pfund J., Schwider J. (2001). Algorithm for Expanding the Dynamic Range of a Shack-Hartmann Sensor by Using a Spatial Light Modulator. Opt. Eng..

[33] Gao Z., Li X., Ye H. (2019). Large Dynamic Range Shack–Hartmann Wavefront Measurement Based on Image Segmentation and a Neighbouring-Region Search Algorithm. Opt. Commun..

[34] Guo H., Korablinova N., Ren Q., Bille J. (2006). Wavefront Reconstruction with Artificial Neural Networks. Opt. Express.

[35] Suárez Gómez S.L., González-Gutiérrez C., García Riesgo F., Sánchez Rodríguez M.L., Iglesias Rodríguez F.J., Santos J.D. (2019). Convolutional Neural Networks Approach for Solar Reconstruction in Scao Configurations. Sensors.

[36] Xu Z., Zhao M., Zhao W., Dong L., Bing X. (2020). Wavefront Reconstruction of Shack-Hartmann Sensorwith Insufficient Lenslets Based on Extreme Learningmachine. Appl. Opt..

[37] He Y., Liu Z., Ning Y., Li J., Xu X., Jiang Z. (2021). Deep Learning Wavefront Sensing Method for Shack-Hartmann Sensors with Sparse Sub-Apertures. Opt. Express.

[38] Li Z., Li X. (2018). Centroid Computation for Shack-Hartmann Wavefront Sensor in Extreme Situations Based on Artificial Neural Networks. Opt. Express.

[39] González-Gutiérrez C., Sánchez-Rodríguez M.L., Calvo-Rolle J.L., de Cos Juez F.J. (2018). Multi-Gpu Development of a Neural Networks Based Reconstructor for Adaptive Optics. Complexity.

[40] González-Gutiérrez C., Santos J.D., Martínez-Zarzuela M., Basden A.G., Osborn J., Díaz-Pernas F.J., de Cos Juez F.J. (2017). Comparative Study of Neural Network Frameworks for the Next Generation of Adaptive Optics Systems. Sensors.

[41] Seifert L., Liesener J., Tiziani H.J. (2003). The Adaptive Shack–Hartmann Sensor. Opt. Commun..

[42] Platt B.C., Shack R. (2001). History and Principles of Shack-Hartmann Wavefront Sensing. J. Refract. Surg..

[43] Southwell W.H. (1980). Wave-Front Estimation from Wave-Front Slope Measurements. JOsA.

[44] Hudgin R.H. (1977). Optimal Wave-Front Estimation. JOsA.

[45] Fried D.L. (1977). Least-Square Fitting a Wave-Front Distortion Estimate to an Array of Phase-Difference Measurements. JOsA.

[46] Cubalchini R. (1979). Modal Wave-Front Estimation from Phase Derivative Measurements. JOsA.

[47] Ríos S., Acosta E., Bará S. (1997). Hartmann Sensing with Albrecht Grids. Opt. Commun..

[48] Noll R.J. (1976). Zernike Polynomials and Atmospheric Turbulence. JOsA.

[49] Liang J., Grimm B., Goelz S., Bille J.F. (1994). Objective Measurement of Wave Aberrations of the Human Eye with the Use of a Hartmann–Shack Wave-Front Sensor. JOSA A.

[50] Rukosuev A., Nikitin A., Belousov V., Sheldakova J., Toporovsky V., Kudryashov A. (2021). Expansion of the Laser Beam Wavefront in Terms of Zernike Polynomials in the Problem of Turbulence Testing. Appl. Sci..

[51] Roddier N.A. (1990). Atmospheric Wavefront Simulation Using Zernike Polynomials. Opt. Eng..

